# Gallium Nitride Semiconductor Resonant Tunneling Transistor

**DOI:** 10.1002/advs.202516334

**Published:** 2026-01-20

**Authors:** Fang Liu, JunShuai Xue, GuanLin Wu, JinYuan Yuan, JiaJia Yao, RenJie Liu, Zhuang Guo, ZeHui Li, HaoRan Hu, WenBo Sun, Cheng Zhao, ChenKai Zhang, XinYan Liu, Kai Zhang, JinCheng Zhang, Yue Hao

**Affiliations:** ^1^ State Key Laboratory of Wide Bandgap Semiconductor Devices and Integrated Technology School of Microelectronics Xidian University Xi'an P. R. China; ^2^ Nanjing Electronic Devices Institute China Electronics Technology Group Corporation Nanjing P. R. China

**Keywords:** high electron mobility transistor, monolithic integrated electronics, negative differential resistance, nitride semiconductor, resonant tunneling diode

## Abstract

Semiconductor devices based on quantum tunneling hold immense promise for developing multi‐valued logic, memory, and oscillators. Recently, two‐terminal gallium nitride (GaN) resonant tunneling diodes (RTDs) have been extensively studied due to their inherent negative differential resistance (NDR) and superior properties of wide bandgap materials. However, conventional GaN RTDs lack current gain, limiting functional modulation and performance improvement. Here, we demonstrate three‐terminal GaN resonant tunneling transistors (RTTs), which comprise an AlN/GaN double‐barrier RTD integrated with a GaN high‐electron‐mobility transistor (HEMT) through epitaxial growth. The GaN RTTs exhibit gate‐tunable NDR behavior through precise control of carrier concentration in the HEMT channel. Remarkably, the series‐connected RTT achieves an NDR voltage span of 4.1 V compared to the 0.41 V span in conventional RTDs, and the parallel‐configured RTT achieves 10 times amplification of peak current by regulating gate voltage. This work provides a feasible approach to tune NDR performance and offers a new opportunity for engineering the functionality of nitride‐based electronics, which is highly expected to alleviate the challenge posed by the saturation of Moore's law and motivate the development of beyond binary logic systems.

## Graphical abstract

Three‐terminal GaN semiconductor resonant tunneling transistors (RTTs), which comprise an double‐barrier AlN/GaN/AlN resonant tunneling diode integrated with a GaN high‐electron‐mobility transistor (HEMT) through epitaxial growth in series and parallel configuraions, respectively. The GaN RTTs exhibit gate‐tunable negative differential resistance behavior through precise control of carrier concentration in the HEMT channel.

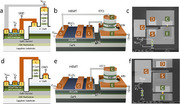

## 1 Introduction

Negative differential resistance (NDR) exhibits non‐ohmic current‐voltage behavior and holds significant potential for versatile device applications, including high‐frequency oscillators, high‐speed switches, cellular neural network, and multi‐level logic circuits [1, 2, 3, 4, 5]. Among the various devices engineered to achieve NDR [5, 6, 7, 8], resonant tunneling diodes (RTDs) stand out due to their exceptionally low junction capacitance and ultrafast switching speeds enabled by quantum tunneling. These properties position RTDs as a leading candidate for next‐generation terahertz oscillators and high‐speed digital circuits [9, 10, 11]. Moreover, RTD is known for its simple device structure and compatibility with mainstream semiconductor processes, offering significant advantages in reducing circuit complexity and enhancing integration [12, 13, 14]. Since its debut in the early 1970s, substantial research efforts have focused on developing high‐performance RTDs and tailoring their NDR characteristics for circuit applications through various different material systems [15, 16, 17]. Recent advances in III‐nitride semiconductor devices have unlocked new possibilities in broadband wireless communication and high‐efficiency energy conversion systems, leveraging their wide bandgap, high critical breakdown field, and exceptional electron saturation velocity [18, 19, 20, 21, 22]. These superior material characteristics enable nitride‐based devices to simultaneously deliver high‐power operation at elevated frequencies, ultrafast switching capabilities in the picosecond regime, and reliable performance across extended temperature ranges, making gallium nitride (GaN) RTD ideal for solid‐state NDR devices.

To address emerging requirements for high‐density storage and high‐performance computing, the NDR feature of GaN‐based RTDs requires enhanced modulation capabilities with high peak current and large peak‐to‐valley voltage span (*ΔV*) [23, 24, 25, 26]. Nevertheless, conventional GaN RTDs, as two‐terminal devices, face fundamental limitations due to their electrical structure, including the absence of current gain and limited modulation capability, which restricts their driving capacity and intrinsic isolation performance. The absence of input‐output isolation in conventional two‐terminal RTDs imposes strict limitations on bias voltage ranges while substantially complicating circuit integration [27, 28, 29]. Furthermore, due to the built‐in strong polarization effect and immature epitaxy technology of nitride quantum well, huge obstacles of the NDR feature tunability of GaN‐based RTDs exist.

To achieve greater modulation versatility and enhanced functionality in GaN RTDs, a promising approach is their monolithic integration of GaN RTDs with three‐terminal GaN electronics on a single platform, facilitating innovative device architectures [28, 29, 30]. The three‐terminal GaN high‐electron‐mobility transistor (HEMT), a cornerstone of microwave power electronics, underpins modern communication technologies. Its operation relies on the gate‐bias modulation of the two‐dimensional electron gas (2DEG) density, which dynamically regulates drain‐source conduction [20]. The monolithic co‐integration of two‐terminal GaN RTDs and three‐terminal GaN HEMTs on a single platform enables a new class of composite three‐terminal resonant tunneling transistors (RTTs) with signal amplification and switching capabilities. In the series‐connected configuration, the composite RTT exhibits gate‐tunable *ΔV* characteristics, where the integrated HEMT functions as a Schottky‐gate‐modulated variable resistor. In the parallel configuration, the RTT output current is the superposition of RTD and HEMT currents. In summary, the composite RTT design provides unprecedented control over NDR properties, including both peak current magnitude and peak‐to‐valley voltage difference. The monolithic integration of GaN HEMTs and GaN RTDs enables compact circuit designs while delivering high driving capability, power gain, and fabrication process compatibility, making it highly suitable for high‐speed digital circuits and high‐frequency, high‐power microwave applications. Additionally, the composite RTT mitigates the constraints imposed by tunneling structure design on circuit performance and expands the diversity of circuit functionality.

In this work, we report three‐terminal GaN semiconductor RTTs exhibiting a gate‐tunable NDR feature with enhanced peak current and peak‐to‐valley voltage span. The developed GaN RTTs are realized by monolithically integrating an AlN/GaN double‐barrier RTD with AlGaN/GaN HEMT. For the three‐terminal GaN RTT operating in parallel topology, the gate voltage regulates the 2DEG concentration in the HEMT channel and tunes the total output current, realizing a linearly modulated peak current in NDR characteristic. By controlling the operation states of GaN HEMT, the output current of the parallel GaN RTT can be amplified up to tenfold compared to that of its constituent RTD. In the series‐connected GaN RTT, the gate voltage applied on the HEMT adjusts the channel resistance and impacts the operation state of RTD, enabling them to dynamically control the NDR characteristics in GaN RTT. The peak‐to‐valley voltage span of the NDR feature in the series‐connected GaN RTT achieves a tenfold improvement over that of the individual GaN RTD by varying gate bias.

## 2 Results and Discussion

### 2.1 Monolithic Integration of GaN RTDs With AlGaN/GaN MIS‐HEMTs

**FIGURE 1:**
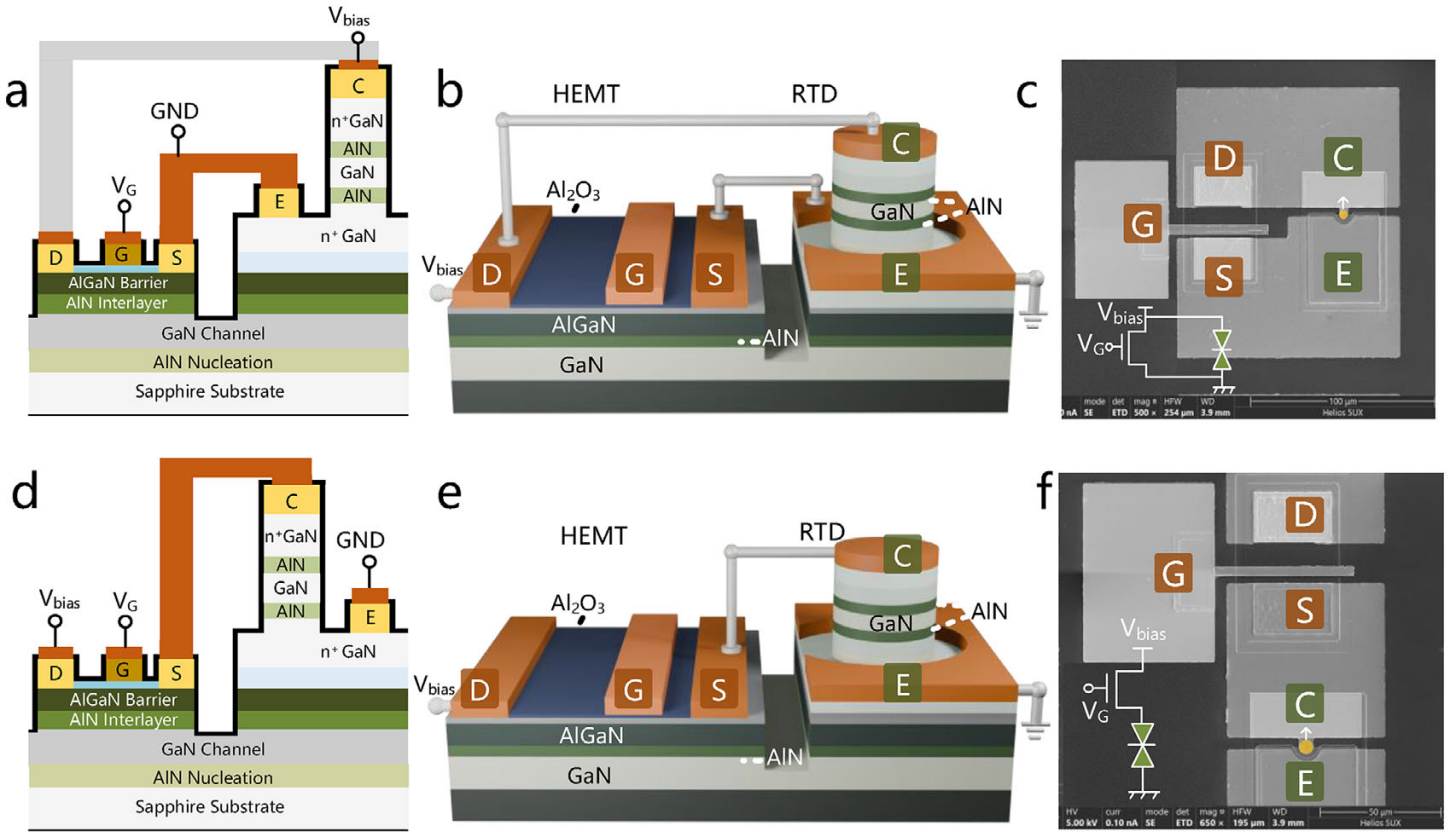
Monolithic integration of AlN/GaN double‐barrier RTD and AlGaN/GaN MIS‐HEMT. (a), Cross‐sectional structure illustration and (b), three‐dimensional device structure of the parallel‐connected GaN RTT. The source, gate and drain electrodes of HEMT are denoted as ‘S’, ‘G’, and ‘D’, respectively, and the collector and emitter Ohmic contact electrodes of RTD are labelled as ‘C’ and ‘E’, respectively. (c), SEM photograph of a fabricated parallel‐connected GaN RTT. Electrical isolation between HEMT and RTD is realized through shallow mesa trench defined by ICP etching. The inset shows the corresponding equivalent circuit diagram. (d), Cross‐sectional view and (e), three‐dimensional device structure of the series‐connected GaN RTT, in combination with the (f) SEM image of the processed series‐connected GaN RTT.

The GaN RTTs were constructed by integrating a three‐terminal AlGaN/GaN MIS‐HEMT with a two‐terminal AlN/GaN double‐barrier RTD. Figure 1 illustrates schematic diagrams and scanning electron microscope (SEM) images of the fabricated parallel‐ and series‐connected GaN RTTs, along with their equivalent circuit diagram and electrodes symbols. Here, the structure of AlN/GaN double‐barrier RTD is placed on top of AlGaN/GaN heterostructures on the *c*‐plane sapphire substrate, which are grown by MBE and MOCVD, respectively, effectively suppressing the lateral leakage currents in GaN HEMTs. Cross‐sectional structure exhibits the epitaxial stacks in detail by focused ion beam‐scanning electron microscope (FIB‐SEM), which is provided in Figure S1. This hybrid epitaxial approach combines the advantages of MOCVD and MBE method, enabling to achieve high electron mobility in AlGaN/GaN heterostructures and high‐quality heterointerface in AlN/GaN/AlN double‐barrier and single quantum‐well (DBSW) [31, 32], because of the critical dependence of resonant tunneling transport on the interface quality and atomic flatness of DBSW heterostructures [33]. The detailed structure of the DBSW active region was revealed by scanning transmission electron microscope (STEM), as shown in Figure S1. To improve current collection efficiency and thus enhance the modulation ability, a ring‐shaped emitter electrode architecture is implemented in the fabrication of RTD, as displayed in Figure S2. A self‐aligned etching process is adopted to precisely define vertical current path of RTD. The consistency between designed and fabricated dimensions is critical for peak current density calculation. We measured the collector dimensions of the RTD array and performed statistical analysis, as shown in Figure S3. Ni metal is used as etching mask to form steep sidewalls, which contributes to the accurate path definition of RTD current as shown in Figure S4. SiN_x_ dielectric layer is deposited on the etching mesa of RTD to suppress sidewall leakage currents.

The performance of AlGaN/GaN HEMTs highly depends on the quality of AlGaN barrier surface in terms of vertical leakage and surface state. Due to the inevitable etching damage during the process of overetching n^+^GaN emitter contact layer on AlGaN barrier, atomic layer deposition (ALD) deposited Al_2_O_3_ dielectric is implemented to form a metal‐insulator‐semiconductor (MIS) gate structure and thus minimize gate leakage currents [34, 35]. Electrical insulation between HEMT and RTD is realized through shallow mesa trench defined by ICP etching. Complete electrical isolation with nearly negligible leakage current is found as shown in Figure S5. The three‐step etching process is integral to the fabrication procedure. We measured the corresponding etching profiles and presented them in Figure S6. In addition, to mitigate the influence of series resistance resulted from the metal‐semiconductor Ohmic contact in source and drain electrodes, the multilayer metal stack of Ti/Al/Ni/Au was annealed utilizing a rapid thermal processing. An illustration of the geometrical formation of device fabrication processes is described in Figure S7. The fabricated AlGaN/GaN HEMTs have a gate length of 3 µm and a gate width of 30 µm with a gate to source distance of 3 µm and a gate to drain distance of 10 µm. Moreover, Ni/Au gate metallization was realized for the HEMT. The gate dimensions of HEMT are carefully designed to enable the saturation region of HEMT to align with the NDR region of RTD. In this work, the monolithic integration between GaN RTD and GaN HEMT is achieved by metal interconnections, as shown in Figure S8. For the parallel topology, as shown in Figure 1a,b, the RTD collector is connected to the drain of the HEMT to form the output terminal, while the emitter of the RTD is connected to the source of the HEMT to form the ground terminal. For the series topology, the source of the HEMT is interconnected with the collector of the RTD, the drain of the HEMT serves as the voltage bias electrode, while the emitter of the RTD is connected to the ground, as illustrated in Figure 1d,e. The plan‐view SEM images of the fabricated series‐ and parallel‐connected GaN RTTs are displayed in Figure 1e,f. Output characteristics of other topologies, such as cascaded parallel‐connected RTTs are also explored in Figure S9. In this work, all the measurements were performed on bare wafer.

### 2.2 AlN/GaN Double‐Barrier RTD Performance

**FIGURE 2:**
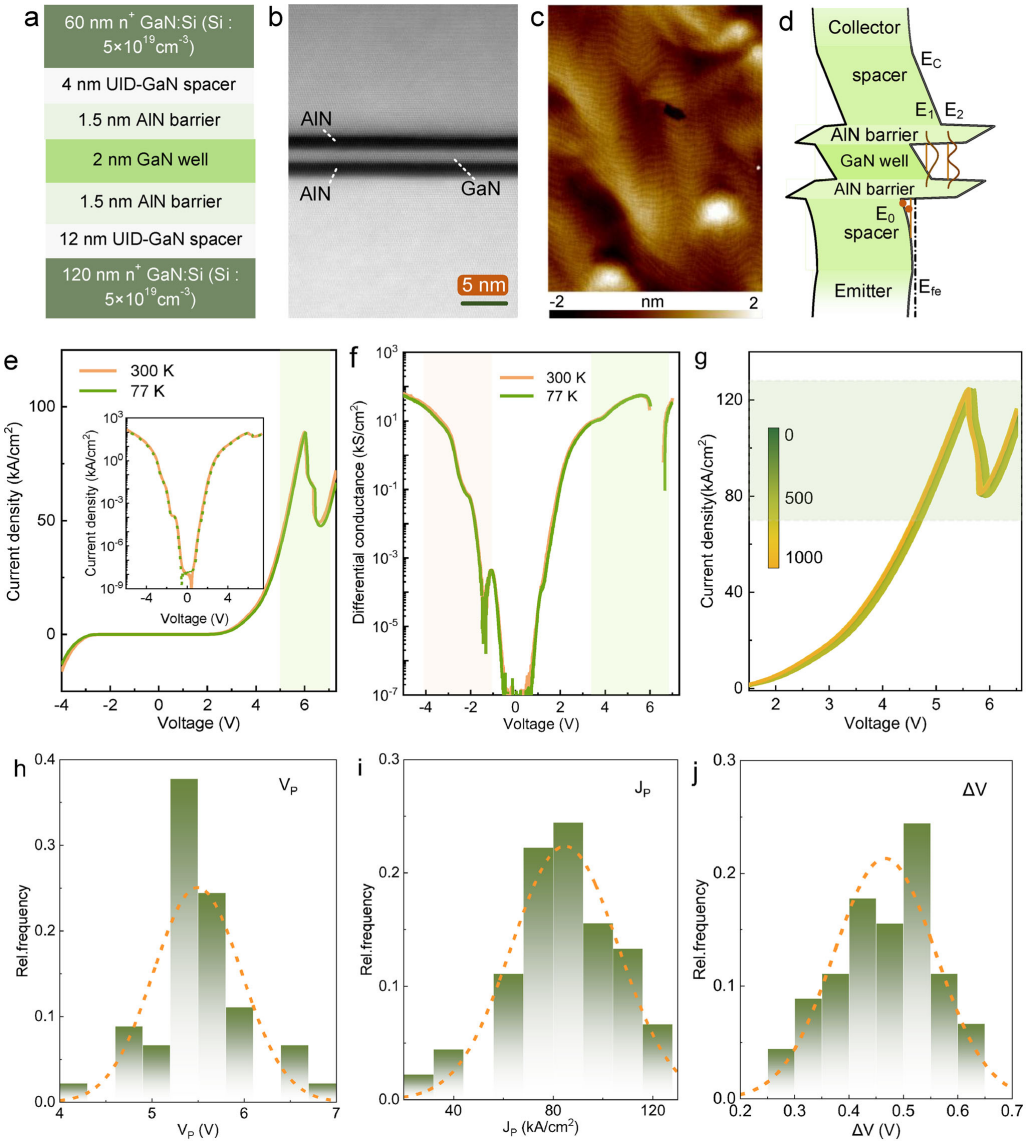
Fabrication and electrical characteristics of the GaN RTDs. (a), Schematic structure of AlN/GaN/AlN double‐barrier RTD grown by PA‐MBE on AlGaN/GaN HEMT. (b), Cross‐sectional HAADF‐STEM image of the double‐barrier single quantum well region of RTD. (c), Surface morphology of the as‐grown RTD material resolved by AFM. (d), Schematic diagram of the energy band structure of the AlN/GaN/AlN RTD at equilibrium. (e), Typical output current‐voltage characteristics of AlN/GaN/AlN double‐barrier RTD with a collector diameter size of 2 µm at 77 and 300 K. The inset illustrates the logarithmic form. (f), Corresponding differential conductance characteristics of double‐barrier RTD. (g), Stability characterization of negative differential resistance in AlN/GaN/AlN double‐barrier RTD device performed at room temperature after a repeatable up‐to‐down bias sweep for continuous 1000 times. Typical statistical analysis of (h), the peak voltage *V*_P_, (i), peak current density *J*_P_ and (j), the difference between peak voltage and valley voltage *ΔV* of double‐barrier RTDs with a collector diameter size of 2 µm.

Figure 2a depicts the schematic structure of AlN/GaN/AlN double‐barrier RTD studied in this work. Here, the core structure of RTD active region is the double‐barrier and single quantum‐well which consists of 2 nm GaN quantum well sandwiched by two 1.5 nm AlN barriers. The large conduction band offset of AlN/GaN heterostructure and ultrathin quantum well induce significant quantum confinement, resulting in a localized and discrete electronic energy spectrum in the GaN quantum well [36]. To obtain low Ohmic contact resistance of collector and emitter electrodes, two degenerately doped n‐type GaN layers are adopted as Ohmic contact layers. Silicon donors are incorporated into these layers with a nominal concentration of 5 × 10^19^ cm^−3^. The performance of collector Ohmic contact is characterized in Figure S10. To suppress dopant diffusion into the active region, undoped GaN spacers are introduced adjacent to each AlN tunneling barrier. The thicknesses of the emitter and collector spacers are 12 and 4 nm, respectively.

Precise control over the thickness of the DBSW layers on the order of the electron wavelength plays a crucial role in resonant tunneling engineering [37]. Besides, smooth and sharp heterointerface of AlN/GaN/AlN DBSW is indispensable to enhance the electron quantum transport in the resonant tunneling cavity by weakening the interface roughness scattering. MBE approach features advantage in precise thickness control on the order of monolayer, and the entire growth of RTD structure maintains metal‐rich regime to achieve a two‐dimensional step‐flow growth mode [38]. Additionally, indium surfactant assisted epitaxy is introduced during the epitaxial growth of AlN/GaN/AlN DBSW, which effectively enables a reduction in surface free energy and diffusion barrier height, facilitating the formation of atomically sharp interface at active region [39]. Figure 2b shows the cross‐sectional scanning transmission electron microscopy (STEM) image of AlN/GaN/AlN DBSW extending over a distance of 35 nm perpendicular to the growth direction. Atomically abrupt interfaces and uniform thickness distribution are resolved in the DBSW layers. Energy‐dispersive X‐ray spectroscopy (EDS) mapping is analyzed in Figure S11, presenting a uniform spatial distribution of aluminum and gallium elements in each layer. As presented in Figure 2c characterized by atomic force microscopy (AFM), an atomically smooth surface featuring step‐flow morphology is revealed with sub‐nanometer root‐mean‐square (RMS) roughness of 0.46 nm over 4 × 5 µm^2^ scan area.

In nitride‐based RTD, the large conduction band discontinuity and the intense electrostatic polarization induce the redistribution of free carriers, resulting in an asymmetric conduction‐band energy distribution in terms of electron accumulation well on the emitter side and depletion region on the collector side, as described in Figure 2d [40]. This pronounced asymmetry in the energy band structure results in notably asymmetric output current‐voltage (*I‐V*) behavior measured at room temperature and 77 K, as exhibited in Figure 2e. From the typical *I‐V* characteristics of fabricated AlN/GaN/AlN double‐barrier RTD with a collector diameter of 2 µm, a distinct N‐type negative differential resistance (NDR) signature is observed under forward bias, which stems from tunneling coupling between the ground quasi‐bound state *E*_1_ in the GaN quantum well and the quasi‐bound state *E*_0_ of accumulated two‐dimensional electron gas (2DEG) in the emitter sub‐well. The obvious NDR feature exhibits a peak current density of 90 kA/cm^2^ at 77 K and 300 K, accompanying with a peak‐to‐valley current ratio (PVCR) of 1.8 at 300 K and 1.88 at 77 K, respectively, indicating suppressed phonon scattering at 77 K. With the decreased temperature, both the peak and valley voltages of the NDR region increase slightly, resulting from the elevation of the quasi‐bound state energy (*E*_1_) induced by temperature reduction [41]. Band diagrams under each resonant condition are provided in Figure S12. Also, electrical performance such as electron concentration profile and electric field distribution of GaN RTD is shown in Figure S21.

As the voltage applied to the RTD collector electrode increases, the *I‐V* curve in logarithmic scale exhibits an exponential current rise, attributed to the effectively modulated transmission probability by the bias voltage [42]. Figure 2f illustrates the corresponding differential conductance curves of output *I‐V* characteristics in logarithmic scale. A less noticed resonance peak emerges at +4 V, which arises from the energy alignment between the 3D electrons presented at Fermi energy level in the emitter side (*E*_fE_) and the ground state (*E*_1_) confined in the quantum well. Under reverse bias voltage at ‐1.3 V, NDR feature emerges at both 77 and 300 K, verified by the breakpoint in the differential conductance curve. Moreover, attenuated carrier tunneling injection between the bound state (*E*_2_) of quantum well and the collector Fermi level (*E*_fC_) forms a differential conductance peak at reverse bias of −2.2 V. The asymmetric tunneling behavior under bipolar bias further highlights the role of polarization electric field in wurtzite nitride‐based electronics. In addition, repeatable and stable NDR characteristics are measured free of any degradation or hysteresis after 1000 consecutive voltage sweeps, as given in Figure 2g.

To comprehensively evaluate the distribution of NDR characteristics in AlN/GaN double‐barrier RTDs, statistical analyses including *V*_P_, *J*_P_, and *ΔV* were performed on 100 randomly selected AlN/GaN double‐barrier RTDs with robust and reliable NDR performance. As displayed in Figure 2h,i, the mean values of *V*_P_ and *J*_P_ are 5.5 V and 84.7 kA/cm^2^, respectively, along with standard deviation of 0.5 V and 21 kA/cm^2^. The DBSW structure in RTD determines the difference between peak voltage and valley voltage *ΔV*, yielding a mean value of 0.47 V and standard deviation of 0.1 V, as shown in Figure 2g. The valley current density (*J*_V_), valley voltage (*V*_V_), and peak‐to‐valley current (*ΔI*) are also statistically analyzed in Figure S13. Given the statistical results, the distribution of these critical parameters in NDR region is highly concentrated and spans a narrow range, benefiting from the uniform and sharp heterointerface of DBSW structures grown by plasma‐assisted molecular beam epitaxy (PA‐MBE). The statistical analysis of RTD parameters provides key guidance for structure design of monolithic integration, especially for the gate width of AlGaN/GaN HEMT, which determines the saturation current of HEMT on the same order of the peak current of GaN RTDs.

### 2.3 AlGaN/GaN MIS‐HEMT Performance

**FIGURE 3:**
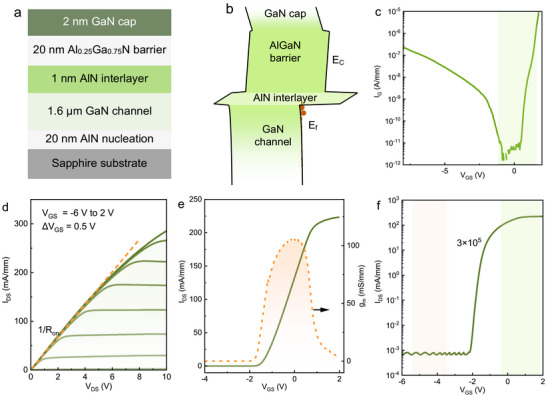
Structure and electrical performance of the AlGaN/GaN HEMT. (a), Schematic structure of AlGaN/GaN heterostructure grown by MOCVD on sapphire substrate. (b), Corresponding equilibrium energy‐band diagram of the AlGaN/GaN heterostructure. (c), Semi‐logarithmic plot of gate leakage current characteristics of AlGaN/GaN MIS‐HEMT. (d), Typical output and (e), transfer characteristics of the fabricated AlGaN/GaN MIS‐HEMT with gate length of 3 µm. (f), The drain current on/off ratio of the corresponding AlGaN/GaN MIS‐HEMT at drain‐source voltage of 6 V.

The AlGaN/GaN heterostructure of MIS‐HEMT is schematically shown in Figure 3a. To reduce the dislocation density of heteroepitaxial GaN epilayer, a 20 nm thick AlN nucleation layer is employed to provide nucleation centers for the GaN crystallites and thus promote the merging and annihilation of dislocation [43]. Unintentionally doped GaN with thickness of 1.6 µm serves as buffer layer and 2DEG channel. A 20 nm thick AlGaN barrier with an aluminum composition of 25% is adopted to form 2DEG in the quantum well of AlGaN/GaN heterostructure, induced by the polarization difference of AlGaN barrier and GaN channel, as indicated in Figure 3b. Besides, 1 nm AlN interlayer is inserted in the heterointerface of AlGaN/GaN heterostructure to improve the electron mobility by shielding alloy disorder scattering and suppressing the interface roughness scattering [44]. To passivate the surface and reduce current collapse, a 2 nm thick GaN cap layer is grown on the AlGaN barrier. This structure design yields an electron density of 0.8 × 10^13^ cm^−2^ with an electron mobility of 2000 cm^2^/V·s at room temperature.

Figure 3c presents the leakage current characteristics of gate electrode in AlGaN/GaN MIS‐HEMT, with a low leakage current density of 2 × 10^−7^ A/mm at gate‐source voltage of ‐8 V, which illustrates that the ALD‐grown Al_2_O_3_ gate dielectric layer effectively restrains the gate leakage, even though the surface of AlGaN barrier is damaged during the etching removal of AlN/GaN double‐barrier RTD structures on top of AlGaN barrier. As presented in Figure 3d, a maximum output current density of 285 mA/mm is obtained at gate‐source voltage of 2 V and drain‐source voltage of 10 V. Here, the relatively low output current density is mainly attributed to the large gate length of 3 µm and large on‐resistance *R*_on_ of 34 mΩ·mm. Absolute current‐voltage characteristics of the device in Figure 3d are discussed in Figure S14, which is an important reference for structure design. Figure 3e plots the transfer characteristics of AlGaN/GaN MIS‐HEMT with a gate length of 3 µm. Typical peak transconductance of 110 mS/mm is revealed at gate‐source voltage of 0 V, and the threshold voltage is determined to be ‐1.4 V by linear extrapolation with the drain current reaching 1 mA/mm. Furthermore, the drain current on/off ratio of the corresponding AlGaN/GaN MIS‐HEMT is 3 × 10^5^ at drain‐source voltage of 6 V, confirming the absence of lateral leakage paths through the n^+^GaN layer in the RTD structure and GaN buffer in the MIS‐HEMT.

### 2.4 Parallel‐Connected GaN RTT

**FIGURE 4:**
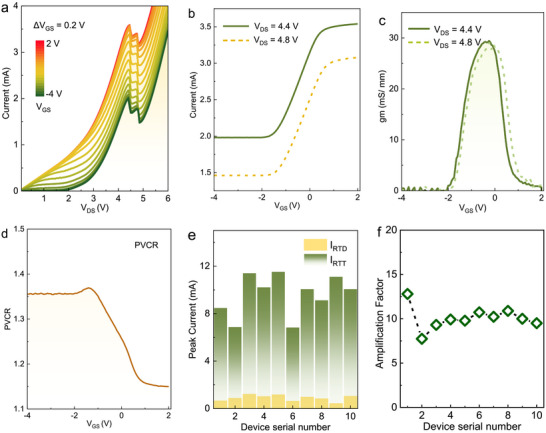
Electrical characteristics of the parallel‐connected GaN RTT. (a), Output characteristics of parallel‐connected GaN RTT with *V*_GS_ biased from −4 to 2 V with 0.2 V steps. (b), Transfer characteristics of the device in a, under *V*_DS_ equals to peak voltage of 4.4 V and valley voltage of 4.8 V in NDR region, respectively. (c), Modulation of *V*_GS_ on the drain current of parallel‐connected GaN RTT under *V*_DS_ equals to peak voltage of 4.4 V and valley voltage of 4.8 V, respectively. (d), Dependence of PVCR on the *V*_GS_ through calculating the ratio of peak current to valley current in the output characteristics under each *V*_GS_. (e), Statistical comparison of output current of parallel‐connected GaN RTT in the off‐state of GaN HEMT with that in the saturation state of GaN HEMT, when the *V*_DS_ equal to peak voltage *V*_P_ in the NDR region of RTT. Under the off‐sate of GaN HEMT in parallel‐connected GaN RTT, the output current of RTT only originates from the GaN RTD. (f), Corresponding amplification factor of output current in GaN RTT compared to that of GaN RTD in **e**.

Next, we evaluated the three‐terminal parallel‐connected GaN RTTs performance. Figure 4a–d present output and transfer characteristics of a typical parallel‐connected GaN RTT with a collector diameter of 2 µm and a gate width of 30 µm. The total output current of parallel‐connected GaN RTT is the sum of the drain‐source current (*I*_DS_) of GaN HEMT and the collector‐emitter current (*I*_CE_) of GaN RTD. To achieve effective modulation of gate‐source voltage (*V*_GS_) on the peak current of parallel‐connected RTT (*I*_RTT_), the NDR region of discrete GaN RTD should overlap with the saturation region of GaN HEMT. As shown in Figure 4a, the RTT exhibits pronounced N‐type NDR and strong dependence of output current on the *V*_GS_ biased from ‐4 to 2 V with 0.2 V steps.

For the *V*_GS_ lower than the threshold voltage of −1.8 V, the GaN HEMT in parallel‐connected GaN RTT is in the off‐state and the RTT behaves as a conventional RTD, where the output current only originates from the GaN RTD and exhibits no dependence on the gate voltage. When the *V*_GS_ is greater than the threshold voltage, the GaN HEMT channel turns on, leading to a rise in drain current. At relatively low drain‐source voltage (*V*_DS_) below 2 V, the transportation of emitter electrons through the RTD cavity is suppressed due to the extremely low transmission coefficient as a result of the misalignment between ground state (*E*_1_) in AlN/GaN/AlN quantum well and 2DEG energy (*E*_0_) [45]. In this status, the RTD remains in a low‐injection state, and the RTT exhibits classical GaN HEMT characteristics, including linear region at low *V*_DS_ and saturation state at higher *V*_DS_. As *V*_DS_ increases, both the peak current and valley currents in the NDR region of the RTD device rise synchronously, confirming the overlap of NDR region in GaN RTD and saturation region of GaN HEMT. Specifically, the peak voltage *V*_p_ of GaN RTD should be larger than the saturation voltage of GaN HEMT, requiring careful structure design of the parallel‐connected GaN RTT. For *V*_GS_ high than 0.4 V, peak and valley currents of GaN RTT no longer vary with the applied *V*_GS_, indicating a constant saturation output current of GaN HEMT. Double‐humped or chair‐like features shown in the NDR region are primarily due to parasitic oscillations caused by the combination of the RTD capacitance and the parasitic inductance in the test circuit.

Figure 4b shows the transfer characteristics of the parallel‐connected GaN RTT, under *V*_DS_ equals to peak voltage of 4.4 V and valley voltage of 4.8 V in NDR region, respectively. Under *V*_DS_ of 4.4 V, the output currents of parallel‐connected GaN RTT for *V*_GS_ lower than −1.8 V and higher than 0.4 V are 2 and 3.53 mA, respectively, corresponding to the off‐state and saturation state of GaN HEMT. Therefore, a gate voltage variation of 2.2 V enables the output current of the parallel‐connected RTT to be modulated by an increment of 1.53 mA, which also represents the change of peak current in the NDR region, as depicted in Figure 4b. Furthermore, the output current of RTT exhibits a linear dependence on *V*_GS_ biased from ‐1.8 to 0.4 V. In addition, the output current at *V*_DS_ of 4.8 V follows a similar trend with the increased *V*_GS_. The modulation of *V*_GS_ on the drain current of parallel‐connected GaN RTT at *V*_DS_ of 4.4 and 4.8 V is further explored in Figure 4c, which yields a typical peak transconductance of 30 mS/mm, highlighting the effective modulation ability of gate voltage in the parallel‐connected RTT. Figure 4d presents the relationship of PVCR with the *V*_GS_, exhibiting an increment of 1.15 to 1.36 when *V*_GS_ decreases from 2 to −1.8 V. Further decreasing *V*_GS_, PVCR nearly remains unchanged, which reflects the output performance of GaN RTD when the 2DEG channel of GaN HEMT is off. Additionally, as shown in Figure 4e, the regulation of *V*_GS_ on the peak current of parallel‐connected GaN RTTs under the off‐state and saturation‐state of the GaN HEMT is statistically analyzed, indicating a dramatical amplification trend. The corresponding amplification factor is extracted in Figure 4f, showing an average ten times current amplification of the parallel‐connected RTT compared with its constituent RTD. The enhanced peak current addresses the challenge of maintaining sufficient drive capability for complex interconnect loads in the pursuit of higher computational speeds in the post‐Moore era, improving the fan‐out capability and providing benefits for applications in ultra‐high‐speed digital logic, multi‐valued logic, and high‐efficiency neuromorphic computing units. The detailed regulation of *V*_GS_ on the valley current can be found in Figure S15. Large‐signal model extraction and electrical performance simulation were carried out and shown in Figure S22. For the parallel topology, the simulated results in Figure S22(c) show the similar variation trend as the experimental data presented in the inset of figure.

### 2.5 Series‐Connected GaN RTT

**FIGURE 5:**
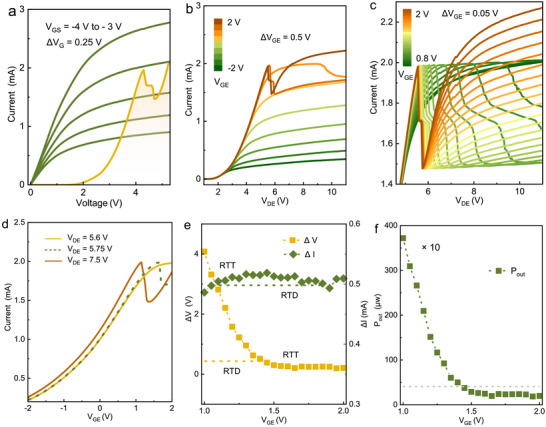
Electrical characteristics of the series‐connected GaN RTT. (a), *I‐V* characteristics of the discrete GaN HEMT under different *V*_GS_ (green lines) and individual GaN RTD (orange line). (b), Output performance of the series‐connected GaN RTT in (a) at *V*_GE_ biased from −2 to 2 V with 0.5 V steps when emitter electrode is grounded. (c), Enlarged output characteristics of device in (b) at *V*_GE_ from 0.8 to 2 V with a voltage step of 50 mV. The *I‐V* curves show evident amplification of *ΔV* in the NDR region. (d), Transfer characteristics of the series‐connected GaN RTT under *V*_DE_ equals to peak voltage of 5.6 V (green line), valley voltage of 5.75 V (yellow dashed line), and voltage in the second PDR of 7.5 V (orange line). The relationship of output current with *V*_GE_ exhibits NDR performance, in which the peak voltage is much lower than that in individual GaN RTT. (e), Variation of *ΔV* and *ΔI* in output characteristics from series‐connected GaN RTT when *V*_GE_ biased from 1 to 2 V. The *ΔV* and *ΔI* for the individual GaN RTD are 0.43 and 0.49 mA, respectively. (f), Dependence of the theoretical maximum output power of the series‐connected RTT on *V*_GE_. The dotted line represents the theoretical maximum output power of the constituent GaN RTD.

The electrical characteristics of the series‐connected GaN RTT are summarized in Figure 5. Here, the collector electrode of GaN RTD is monolithically integrated with the source terminal of GaN HEMT, which serves as driver in the topology of series‐connected GaN RTT. Electrons from emitter undergo resonant tunneling through the AlN/GaN/AlN DBSW structure of GaN RTD, resulting in NDR current. Then, the NDR current flows through the 2DEG channel of GaN HEMT and is tuned by the Schottky gate voltage [46]. To distinguish their individual contributions, the discrete components of RTD and HEMT were separately characterized as shown in Figure 5a, providing a baseline for analyzing the performance of series‐connected GaN RTT. The *I‐V* characteristic of individual GaN RTD exhibits a pronounced NDR feature, with an *I*_P_ of 2 mA at *V*_P_ of 4.3 V together with a PVCR value of 1.34. Typical current‐voltage characteristics of GaN RTDs are presented in Figure S16. When the *V*_GS_ of GaN HEMT is biased at −4 to −3 V in 0.25 V voltage steps, the saturation region of GaN HEMT overlaps with the NDR region of GaN RTD, as depicted in Figure 5a. Additional transfer characterization of the GaN HMET is presented in Figure S17.

Figure 5b presents the output characteristics of the series‐connected GaN RTT under the drain‐emitter voltage (*V*_DE_). First, we explored the operation mechanism of series‐connected GaN RTT at different gate‐emitter voltage (*V*_GE_). When *V*_GE_ is below 1 V, at relatively low *V*_DE_, RTD locates under low injection state and effectively blocks the transport of carriers, behaving as a non‐conducting diode and series resistance [47]. As *V*_DE_ increases, the blocking effect of the GaN RTD barriers on the carrier injection diminishes, and 2DEG channel of GaN HEMT turns on and dominates the output current. Notably, due to the series configuration, the knee voltage of the series‐connected GaN RTT is higher than that of the individual GaN HEMT. When the *V*_GE_ exceeds 1 V, the 2DEG concentration in the GaN HEMT channel rapidly rises, leading to an increased saturation output current and a reduced channel resistance. With further increasing *V*_GE_, the channel resistance of GaN HEMT continuously declines, the proportion of *V*_DE_ applied on GaN RTD increases and the resonant tunneling transport is triggered in GaN RTD when the ground state in DBSW aligns with the 2DEG level in emitter sub‐well, yielding a distinct NDR feature. Figure 5c enlarges the output characteristics of series‐connected GaN RTT at *V*_GE_ from 0.8 to 2 V with a voltage step of 50 mV, showing evident amplification of *ΔV* in the NDR region. It is indicated that the peak voltage in NDR region shifts downward during the *V*_GE_ increases.

As given in Figure 5d, the transfer characteristics of the series‐connected GaN RTT exhibit NDR performance under *V*_DE_ of 7.5 V, featuring a peak voltage of 1.1 V which is significantly lower than that of individual GaN RTD [48]. Transfer characteristics show similar feature for *V*_DE_ biased at NDR and the second positive differential resistance (PDR) region, a consequence of resonant tunneling of GaN RTD. The corresponding transconductance performance is provided in Figure S18. Here, the NDR performance obtained in transfer characteristics is different from that achieved in GaN RTD. The former is realized through gate modulation of lateral 2DEG channel current, while the latter embodies the conventional vertical resonant tunneling in AlN/GaN/AlN DBSW. Notably, the *ΔV*_RTT_ of the NDR feature in series‐connected GaN RTT undergoes significant adjustment when *V*_GE_ biased from 1 to 2 V. The individual GaN RTD exhibits a *ΔV*_RTD_ of 0.43 V, while the series‐connected GaN RTT achieves a significantly enhanced *ΔV*_RTT_ of 4.1 at *V*_GE_ of 1 V, representing a 10 times improvement compared to that of the individual GaN RTD, as demonstrated in Figure 5e. This amplification arises from variation of channel resistance under modulation of *V*_GE_. Overall, HEMT operating in saturation state acts as a controlled variable resistor in our series RTD‐HEMT circuit. The reduced *V*_GS_ leads to a significant increase in the access channel resistance of the HEMT. Therefore, a higher *V*_DE_ is required for the RTD to reach the valley point. This is reflected a larger *ΔV* on the output curve *I*_DE_‐*V*_DE_. Particularly, while the *ΔI*_RTT_ is almost identical to that of the discrete GaN RTD as shown in Figure 5c, the series‐connected GaN RTT exhibits a prominent advantage to achieve exceptionally broad and flat NDR region. This unique property facilitates the enhancement of the negative‐resistance oscillator performance, amplifying the maximum output power by a factor of 10 relative to a single RTD device, as compared in Figure 5f [49]. For the series topology, the simulated results in Figure S22d show the similar trend with the experimental results shown in its inset. The plateau phenomenon observed in the measured curves is attributed to parasitic oscillations. The broadening of *ΔV* allows the oscillator to directly drive load components such as antennas or mixers. Concurrently, the expanded operating voltage window significantly reduces sensitivity to bias fluctuations and temperature drift, thereby meeting the requirements for high‐precision radar and spectrally pure signal sources. The broadening of *ΔV* directly provides greater potential for output voltage swing, enhancing the oscillator's output power. A schematic diagram of an RTD‐based oscillator circuit and oscillation spectrum for NDR devices with different *ΔV* are simulated and compared in Figure S23.

## 3 Conclusion

In summary, we investigated an effective approach to modulate the NDR characteristics in GaN quantum tunneling devices through GaN RTT by monolithically integrating an AlN/GaN double‐barrier RTD with an AlGaN/GaN HEMT. When the GaN RTT operates in parallel topology, the total output current consists of the drain‐source current of GaN HEMT and the collector‐emitter current of GaN RTD. By adjusting the NDR region of discrete GaN RTD to overlap with the saturation region of GaN HEMT, gate voltage enables to effectively modulate the peak current of parallel‐connected RTT. The parallel‐connected RTT achieves an average ten times current amplification compared to that of its constituent RTD. When the three‐terminal RTT operates in series topology, NDR feature results from emitter electrons undergoing resonant tunneling in the AlN/GaN/AlN DBSW of the integrated GaN RTD and is tuned by the Schottky gate voltage applied on the 2DEG channel of GaN HEMT. The transfer characteristics of series‐connected GaN RTT exhibit NDR phenomenon through modulation of channel current in GaN HEMT. The series‐connected RTT achieves 10 times amplification of the NDR voltage span, yielding a significant enhancement in maximum output power compared to individual RTD devices. This work pioneers a novel approach to achieving nitride‐based high‐speed electronic devices with tunable NDR characteristics. Furthermore, this demonstrated device platform facilitates the development of multifunctional integrated electronic systems.

## 4 Experimental Section

### 4.1 Epitaxial Growth of Nitride Epilayers

First, GaN heterostructures were grown on 4‐inch *c*‐plane sapphire substrates by metal‐organic chemical vapor deposition (MOCVD). Here, trimethylaluminum (TMAl), trimethylgallium (TMGa) and ammonia (NH_3_) were used as the metal‐organic precursors of Al, Ga and anion source, respectively. Prior to growth, nitridation was performed at 1100°C for 10 min in an NH_3_ ambient after annealing the substrate in hydrogen for 10 min at 1100°C. The growth was initiated with a 10 nm AlN nucleation layer grown at 620°C followed by 10 nm AlN layer at 1100°C. Subsequently, 1.6 µm nominally undoped GaN channel was grown at 1020°C followed by about 1 nm AlN interlayer and 20 nm AlGaN barrier with an aluminum composition of 25%, where the AlN interlayer was adopted to enhance the mobility of 2DEG. Finally, a 2 nm GaN cap layer was deposited on the AlGaN barrier.

Second, the MOCVD‐grown AlGaN/GaN heterostructures were cleaned using standard wet chemical cleaning procedures prior to loading into the ultrahigh vacuum (UHV) chamber of PA‐MBE. The cleaning procedures consisted of initial organic contaminant removal using acetone and isopropanol solvent rinses, followed by surface oxide elimination through hydrochloric acid solution. Then, the epitaxial AlGaN/GaN wafer was loaded into the load lock chamber to thermally degas and subsequently into the preparation chamber. Next, the wafer was transferred into the growth chamber and cleaned using gallium deposition and desorption approach. After finishing these, a clean and fresh surface was obtained for regrowth of GaN RTD epilayers by PA‐MBE.

Third, AlN/GaN/AlN double‐barrier resonant tunneling diode (RTD) active layers were grown by PA‐MBE on MOCVD‐grown AlGaN/GaN heterostructures. All of the RTD structures were grown continuously at a substrate temperature of 675°C throughout the entire growth, under metal‐rich growth regime to obtain a two‐dimensional step‐flow growth mode. Here, the growth was performed at a pressure of 2 × 10^−6^ Torr under a nitrogen source flow rate of 0.6 sccm through the radio frequency plasma source with 375 W power. The beam equivalent pressure adopted in the epitaxy was 2 × 10^−7^ Torr for gallium and 0.8 × 10^−7^ Torr for aluminum. First, 120 nm heavily doped GaN epilayer with a silicon dopant concentration of 5 × 10^19^ cm^−3^ was deposited as the Ohmic contact layer on the emitter side, followed by double‐barrier single‐quantum consisting of a 2 nm‐thick quantum well sandwiched by two 1.5 nm‐thick AlN barriers. Finally, 60 nm n‐type GaN Ohmic contact layer was deposited on the collector side with the same dopant concentration as the emitter side. 12 and 4 nm thick undoped GaN spacer layers were inserted between the AlN barrier layers and the Ohmic contact layers on the emitter side and collector side, respectively. Shutter status of MBE during the growth process of GaN RTD is illustrated in Figure S19. The X‐ray diffraction (XRD) pattern of the as‐grown sample is displayed in Figure S20.

### 4.2 Device Fabrication Process

First, define the electrodes of AlGaN/GaN HEMT. First, the epitaxial wafer underwent thorough organic and inorganic cleaning, a process similar to the above‐mentioned epitaxial cleaning procedure. The epitaxial wafer was etched from n‐type GaN Ohmic contact layer on the collector side of RTD down to the AlGaN barrier of GaN HEMT by inductively coupled plasma (ICP) etching to form the mesa of the HEMT, using a reactive gas mixture consisting of 20 sccm chlorine (Cl_2_) and 8 sccm boron trichloride (BCl_3_) at 250 W RF power and 40 W bias power. After that, a multilayer metal stack composed of Ti/Al/Ni/Au (20/120/45/55 nm) was evaporated as source and drain electrodes of GaN HEMT through electron beam evaporation (EBE). Then, ICP etching was employed to etch from the AlGaN barrier surface down to the GaN channel, resulting in an electrical insulation trench between the RTD and HEMT regions. Following that, the multilayer metal stack of Ti/Al/Ni/Au was annealed utilizing a rapid thermal processing (RTP) at 830°C for 30 s in a pure nitrogen (N_2_) ambient environment to achieve low contact resistance of source and drain electrodes of GaN HEMT.

Second, evaporate the collector and emitter electrodes of AlN/GaN/AlN double‐barrier RTD. Variable‐sized mesa patterns of GaN RTDs were defined by traditional photolithography, followed by the evaporation of Ti/Au/Ni (20/80/10 nm) metal stack using EBE. After that, a self‐aligned ICP etching process was applied to precisely define the vertical current path of GaN RTD, using the Ni metal as the etch mask. The epitaxial wafer of defined GaN RTD region was etched from n‐type GaN Ohmic contact layer on the collector side down to the n‐type GaN Ohmic contact layer on the emitter side. Then, a semi‐enclosed pattern of emitter electrode surrounding the RTD mesa was defined, followed by the evaporation of Ti/Au (20/80 nm) bilayer.

Third, fabricate the gate electrode of AlGaN/GaN HEMT, deposit passivation dielectric and open vias on the electrodes. The Al_2_O_3_ (10 nm) gate dielectric of GaN HEMT was deposited by plasma‐enhanced atomic layer deposition (PEALD) at 250 °C using trimethylaluminum (TMA) and oxygen (O_2_) as precursors. Subsequently, the gate electrode of GaN HEMT was patterned and evaporated with Ni/Au (50/150 nm) stack by EBE. A 250 nm thick silicon nitride (SiN_x_) layer was deposited as passivation and electrical insulation layers by plasma‐enhanced chemical vapor deposition (PECVD) at 300 °C, using precursor gases of silane (SiH_4_), ammonia (NH_3_), and argon (Ar). Finally, vias were formed in SiN_x_ layer by exposing the electrodes’ metals using RIE at 150 W power with 5 sccm O_2_ and 25 sccm CHF_3_ gases.

Finally, metal interconnection of AlGaN/GaN HEMT and AlN/GaN/AlN RTD electrodes. For series‐connected GaN RTT configuration, the source electrode of AlGaN/GaN HEMT and collector electrode of AlN/GaN/AlN RTD were interconnected through Ti/Au (20/100 nm) stack. In the parallel GaN RTT architecture, the source electrode of AlGaN/GaN HEMT and emitter of AlN/GaN/AlN RTD were bonded as the ground terminal, while the drain electrode of AlGaN/GaN HEMT and collector electrode of AlN/GaN/AlN RTD were interconnected to form the output terminal. The detailed fabrication process has been illustrated in Figure S5.

### 4.3 Material and Device Characterization

The surface morphology of the as‐grown material and the etched wafer was resolved by atomic force microscopy (AFM) using a Bruker ICON system. Both (002) symmetric and (102) asymmetric rocking curves were measured using a high‐resolution X‐ray diffraction (HRXRD) performed on a Bruker D8 system equipped with a Cu Kα radiation source. The specimen for scanning transmission electron microscope (STEM) was prepared using in‐situ focused ion beam (FIB) lift‐out technique in a Thermo Scientific Helios G4 HX UC dual‐beam FIB‐SEM system, where the sample surface was protected by ion‐beam‐deposited platinum (Pt) to prevent ion beam damage. Sub‐angstrom‐resolution aberration‐corrected high‐angle annular dark‐field (HAADF) STEM imaging measurements were acquired using a Thermo Scientific Themis Z spherical TEM instrument operating at 200 kV. The process monitoring of the GaN RTT device fabrication was obtained using a Thermo Scientific Helios 5UX scanning electron microscope (SEM) operating in secondary electron (SE) mode at an accelerating voltage of 5 kV. The etch step depth profile was examined using a Bruker Dektak surface profiler with a 2‐µm‐radius tip under 3‐mg contact force.

The output *I‐V* characteristics measurements were carried out using a semiconductor parameter analyzer (Keysight B1500A). Temperature‐dependent electrical characterization was performed using a Lakeshore CRX‐4K cryogenic probe station equipped with a closed‐cycle helium refrigeration system. The device under test (DUT) was mounted on a gold‐plated copper sample holder with indium foil for optimal thermal contact.

### 4.4 Energy Band Structure Modelling and Analysis

The Poisson‐Schrödinger equation and the nonequilibrium Green's function (NEGF) approach were used to calculate the energy band structure of AlN/GaN/AlN double‐barrier RTD. The band structure of GaN HEMTs was determined by self‐consistently solving the Schrödinger equation and Poisson equation, incorporating polarization‐induced charges and 2DEG density via Fermi‐Dirac statistics.

## Conflicts of Interest

The authors declare no conflict of interest.

## Supporting information




**Supporting file**: advs73421‐sup‐0001‐SuppMat.docx

## Data Availability

The data that support the findings of this study are available from the corresponding author upon reasonable request.
