# Supplementary material for: Gallium Nitride Semiconductor Resonant Tunneling Transistor
Source: Adv Sci (Weinh). 2026 Jan 20;13(13):e16334. doi: 10.1002/advs.202516334 (PMC12955862; doi:10.1002/advs.202516334)
Supplement: Supplementary file 1 — Supporting file: advs73421‐sup‐0001‐SuppMat.docx [file ADVS-13-e16334-s001.docx]

**Gallium nitride semiconductor resonant tunneling transistor**

*Fang Liu,^1^ JunShuai Xue,^1,^*^)^ GuanLin Wu,^1^ JinYuan Yuan,^1^ JiaJia Yao,^1^ RenJie Liu,^1^ Zhuang Guo,^1^ZeHui Li, ^1^ HaoRan Hu,^1^ WenBo Sun,^1^ Cheng Zhao,^1^ ChenKai Zhang,^1^XinYan Liu,^1^Kai Zhang,^2^ JinCheng Zhang,^1,^*^)^ and Yue Hao^1^*

***Supporting information1***

***Table of contents***

**Supplementary Fig. S1. Scanning electron microscope (SEM) images of the monolithically integrated GaN resonant tunneling transistor (RTT).**

**Supplementary Fig. S2. SEM images of the discrete AlN/GaN/AlN double-barrier resonant tunneling diode (RTD).**

**Supplementary Fig. S3. SEM image of fabricated GaN RTD array and statistical analysis of their collector diameters.**

**Supplementary Fig. S4. Three-dimension atomic force microscopy (AFM) topography of the GaN RTD array after etching collector mesa.**

**Supplementary Fig. S5. Electrical isolation performance between GaN HEMT and GaN RTD.**

**Supplementary Fig. S6. Etch depth distribution of multiple steps for the fabricated GaN RTT structure.**

**Supplementary Fig. S7. Key fabrication steps for monolithically integrated GaN RTTs.**

**Supplementary Fig. S8. Enlarged SEM image of the GaN RTTs.**

**Supplementary Fig. S9. Output characteristics of cascaded parallel-connected GaN RTTs topology.**

**Supplementary Fig. S10. Collector Ohmic contact characterization of GaN RTD using circular transmission line method (CTLM).**

**Supplementary Fig. S11.** **Energy-dispersive X-ray spectroscopy (EDS) mapping analysis of double-barrier and single quantum well (DBSW) region in GaN RTD.**

**Supplementary Fig. S12. Band diagram of AlN/GaN/AlN double-barrier RTD under resonant conditions.**

**Supplementary Fig. S13.** **Statistical analysis of the valley current density (*J*_V_), valley voltage (*V*_V_), and peak-to-valley current difference (*ΔI*) of GaN RTDs.**

**Supplementary Fig. S14.** **Typical absolute output current characteristics of discrete GaN HEMT.**

**Supplementary Fig. S15.** **Electrical characteristics of the parallel-connected GaN RTT.**

**Supplementary Fig. S16.** **Typical absolute output current characteristics of discrete AlN/GaN double-barrier RTD.**

**Supplementary Fig. S17. Transfer characteristics of the discrete AlGaN/GaN HEMT in the series-connected GaN RTT as shown in Figure 5a.**

**Supplementary Fig. S18. Transconductance characteristics of the series-connected GaN RTT.**

**Supplementary Fig. S19.** **Shutter status of molecular beam epitaxy (MBE) during the growth of AlN/GaN/AlN double-barrier RTD.**

**Supplementary Fig. S20. X-ray diffraction (XRD) rocking curves of GaN grown on free-standing GaN substrate by molecular beam epitaxy.**

**Supplementary Fig. S21.** **Electrical performance of AlN/GaN/AlN double-barrier RTD.**

**Supplementary Fig. S22.** **Simulated** **electrical performance of GaN RTTs.**

**Supplementary Fig. S23.** **Schematic diagram of an RTD-based oscillator circuit and oscillation spectrum.**

**Supplementary Fig. S1. Scanning electron microscope (SEM) images of the monolithically integrated GaN resonant tunneling transistor (RTT).** (**a**) Global overview SEM image of RTTs. (**b**) SEM image of a series-connected GaN RTT prepared for focused ion beam (FIB) milling. SEM image of the series-connected GaN RTT structure including (**c**) GaN HEMT region, (**d**) mesa isolation region, (**e**) and GaN RTD region. The inset in (**e**) shows the AlN barrier in GaN RTD and AlGaN barrier in GaN HEMT.

**Supplementary Fig. S2. SEM images of the discrete AlN/GaN/AlN double-barrier resonant tunneling diode (RTD).** Close-up SEM image of the operation region of RTD, showing a circular collector electrode and a semicircular ring-shaped emitter electrode.

**Supplementary Fig. S3. SEM image of fabricated GaN RTD array and statistical analysis of their collector diameters.** SEM image of collector array with a diameter of 2 μm is captured and measured, demonstrating the precise definition of collector electrode achieved by the fabrication process, which is a critical factor to accurately calculate current density. The average diameter of collector electrodes is 2 μm with a standard deviation of 0.024 μm, reflecting the highly control ability of device processing.

**Supplementary Fig. S4. Three-dimension atomic force microscopy (AFM) topography of the GaN RTD array after etching collector mesa.** The topography shows steep sidewalls, contributing to accurate path definition of the output current.

**Supplementary Fig. S5. Electrical isolation performance between GaN HEMT and GaN RTD.** Current-voltage characteristics between drain and emitter electrodes demonstrate complete electrical isolation with nearly negligible leakage current.

**Supplementary Fig. S6. Etch depth distribution of multiple steps for the fabricated GaN RTT structure.** Step 1 represents the etching step from n-type GaN Ohmic contact layer on the collector side of GaN RTD down to the AlGaN barrier of GaN HEMT, forming the mesa of the GaN HEMT. Step 2 shows the etching step from the AlGaN barrier surface down to the GaN channel of GaN HEMT, behaving as electrical insulation trench between the GaN RTD and GaN HEMT regions. Step 3 displays the etching step from n-type GaN Ohmic contact layer on the collector side of GaN RTD down to the n-type GaN Ohmic contact layer on the emitter side of GaN RTD, defining GaN RTD mesa.

**Supplementary Fig. S7. Key fabrication steps for monolithically integrated GaN RTTs.**

① Completely clean the surface of the epitaxial wafer using organic and inorganic solution.

② Etch the wafer from n-type GaN Ohmic contact layer on the collector side of GaN RTD down to the AlGaN barrier of GaN HEMT by inductively coupled plasma (ICP) etching, forming the mesa of the GaN HEMT.

③ Evaporate multilayer metal stack of Ti/Al/Ni/Au as source and drain electrodes by electron beam evaporation (EBE).

④ Etch the AlGaN barrier surface down to the GaN channel as electrical isolation trench between the GaN RTD and GaN HEMT regions by ICP. Subsequently, the evaporated multilayer metal stack is annealed utilizing rapid thermal processing (RTP).

⑤ Evaporate the collector metal of AlN/GaN/AlN double-barrier RTD. Then, etch the n-type GaN Ohmic contact layer on the collector side down to the n-type GaN Ohmic contact layer on the emitter side to form GaN RTD mesa, using self-aligned ICP process with the Ni metal as the etch mask.

⑥ Evaporate emitter metal surrounding the GaN RTD mesa.

⑦ Deposit gate dielectric using atomic layer deposition (ALD) and evaporate Ni/Au metal as gate electrode of AlGaN/GaN HEMT.

⑧ Deposit SiNₓ layer as passivation layer and electrical insulation layers by plasma-enhanced chemical vapor deposition (PECVD).

⑨ Etch SiNₓ layer on the electrodes metal using reactive ion etching (RIE) to form vias.

⑩ Evaporate metal interconnects of AlGaN/GaN HEMT and AlN/GaN/AlN RTD electrodes.

**Supplementary Fig. S8. Enlarged SEM image of the GaN RTTs.** SEM photographs of fabricated (**a**) parallel-connected GaN RTT and (**b**) series-connected GaN RTT with marked electrode name. The insets illustrate the equivalent circuit diagram of GaN RTTs.

**Supplementary Fig. S9.** **Output characteristics of cascaded parallel-connected GaN RTTs topology.** (a) Output characteristics of the series unit when the gate voltage of the load device *V*_GL_ is fixed at -6 V (OFF state) while sweeping the gate voltage *V*_GD_ of the driving RTT. (b) Output characteristics of the series unit when the gate voltage *V*_GD_ of the driving RTT is fixed at -6 V (OFF state) while sweeping the gate voltage *V*_GL_ of the load RTT. (c) Output characteristics of the individual parallel-connected RTTs in cascaded parallel-connected GaN RTTs topology. These characterizations are critical for demonstrating the applicability of GaN RTTs in inverter circuits.

**Supplementary Fig. S10.** **Collector Ohmic contact characterization of GaN RTD using circular transmission line method (CTLM).** (**a**) *I-V* characteristics of test pads with varying spacings at room temperature, with total resistance *R*_T_ determined by linear fitting. (**b**) Plot of ln(R/r) versus total resistance *R*_T_. *R* is the radius of the outer ring and *r* is the radius of the inner ring. Linear fitting yields a slope of 48 and y-intercept of 3.1, corresponding to a collector contact specific resistance of 3.1×10^-5^ Ω·cm².

**Supplementary Fig. S11. Energy-dispersive X-ray spectroscopy (EDS) mapping analysis of double-barrier and single quantum well (DBSW) region in GaN RTD.** The EDS elemental mapping of aluminum (red) and gallium (blue) for epitaxial active region illustrates an obvious contrast between AlN barriers and GaN well and presents a uniform spatial distribution of aluminum and gallium elements in each layer.

**Supplementary Fig. S12. Band diagram of AlN/GaN/AlN double-barrier RTD under resonant conditions.** (**a**) Resonant tunneling when the quasi-bound energy (*E*_0_) of two-dimensional electron gas (2DEG) accumulated in the emitter sub-well aligns with the ground state *E*_1_ in GaN well. (**b**) The resonant tunneling injection of carriers from the Fermi energy level (*E*_fE_) in the emitter side into the discrete level *E*_1_ in GaN well. (**c**) The alignment of ground-state energy (*E*_1_) in the GaN quantum well with the Fermi level (*E*_fC_) on the collector side.

**Supplementary Fig. S13.** **Statistical analysis of the valley current density (*J*_V_), valley voltage (*V*_V_), and peak-to-valley current difference (*ΔI*) of GaN RTDs.** Typical statistical analysis of (**a**) the *J*_V_ and (**b**) the *V*_V_ of AlN/GaN/AlN double-barrier RTDs with a collector diameter size of 2 µm. The mean values of *J*_V_ and *V*_V_ are 80.7 kA/cm^2^ and 6 V, respectively, along with standard deviation of 23 kA/cm^2^ and 0.5 V. The dashed lines are Gaussian fittings. These parameters in NDR region show a highly concentrated distribution. (**c**) Statistical analysis of the peak-to-valley current difference *ΔI* of GaN RTDs with a collector diameter size of 2 µm. The mean values and standard deviation of *ΔI* are 0.68 mA and 0.3 mA, respectively.

**Supplementary Fig. S14.** **Typical absolute output current characteristics of discrete GaN HEMT.** (**a**) Typical output performance of discrete GaN HEMT analyzed in **Figure 3d** with *V*_GS_ biased from -6 V to 2 V with 0.5 V steps. (**b**) Transfer characteristics of the device in (**a)**, under *V*_DS_ of 6 V. A maximum output current of 6.7 mA is obtained at *V*_GS_ of 2 V and *V*_DS_ voltage of 6 V. These analysis of discrete GaN HEMT provides significant guidance for structure design of monolithic integration, especially for the gate width of AlGaN/GaN HEMT.

**Supplementary Fig. S15.** **Electrical characteristics of the parallel-connected GaN RTT.** (**a**) Statistical comparison of valley current (*I*_VRTD_) of parallel-connected GaN RTT in the off-state of GaN HEMT with that (*I*_VRTT_) in the saturation state of GaN HEMT, when the *V*_DS_ equal to valley voltage *V*_V_ in the NDR region of GaN RTT. Under the off-sate of GaN HEMT in parallel-connected GaN RTT, the output current of RTT only originates from the GaN RTD. (b) Output characteristics of parallel-connected GaN RTT with *V*_GS_ biased from -4 V to 2 V with 0.25 V steps. Relatively large peak voltage compared to **Figure 4a** presents a broader saturation region.

**Supplementary Fig. S16.** **Typical absolute output current characteristics of discrete AlN/GaN double-barrier RTD.** Typical output current-voltage characteristics of AlN/GaN/AlN double-barrier RTD with a collector diameter size of 2 µm. Absolute peak current of GaN RTD is designed to match the saturation output current of GaN HEMT on the same order.

**Supplementary Fig. S17.** **Transfer characteristics of the discrete AlGaN/GaN HEMT in the series-connected GaN RTT as shown in Figure 5a.** Transfer performance of the discrete GaN HEMT at *V*_GS_ biased from -8 V to 2 V with 0.05 V steps when source electrode is grounded.

Typical peak transconductance of 120 mS/mm is revealed at *V*_GS_ of -1.7 V. The threshold voltage is determined to be -4.2 V by linear extrapolation with the drain current reaching 1 mA/mm.

**Supplementary Fig. S18.** **Transconductance characteristics of the series-connected GaN RTT.** Transconductance characteristics of the series-connected GaN RTT corresponding to **Figure 5d** under *V*_DE_ equals to peak voltage of 5.6 V (green line), valley voltage of 5.75 V (yellow dashed line), and voltage in the second PDR of 7.5 V (orange line). Negative transconductance exhibits at *V*_GE_ of 1.1 V which is significantly lower than the applied voltage required for an individual GaN RTD.

**Supplementary Fig. S19. Shutter status of molecular beam epitaxy (MBE) during the growth of AlN/GaN/AlN double-barrier RTD.** Shutter status of gallium (Ga), aluminum (Al), indium (In) effusion cells, and nitrogen (N_2_) RF plasma source for the growth of AlN/GaNAlN double-barrier RTD. The shutter of the indium effusion cell was open during the growth of the AlN/GaN/AlN quantum well, resulting in the formation of a metallic layer. This method enables to reduce the sticking coefficient and diffusion barrier height of aluminum adatoms, thus enhancing the surface diffusion length.

**Supplementary Fig. S20.** **X-ray diffraction (XRD) rocking curves of GaN grown on free-standing GaN substrate by molecular beam epitaxy.** XRD 2*θ* scan across the symmetric (002) and asymmetric (102) reflection of GaN grown on free-standing GaN substrate, confirming the excellent quality of homoepitaxial GaN.

**Supplementary Fig. S21.** **Electrical performance of AlN/GaN/AlN double-barrier RTD.** (**a**) Conduction-band diagram and electron concentration profile of RTD at equilibrium state (blue). (**b**) Conduction-band diagram and electron concentration profile of RTD under forward bias when 2DEG energy level *E*_0_ aligns with the ground state *E*_1_ in quantum well. (**c**) Electric field diagram at equilibrium state and (**d**) resonant tunneling state when *E*_0_ aligns with *E*_1_.

**Supplementary Fig. S22.** **Simulated** **electrical performance of GaN RTTs.** (**a**) Simulated DC output characteristics of the HEMT device. (**b**) The large-signal model of the RTD by fitting the measured *I-V* curves. (**c**) Simulated output characteristics of the HEMT/RTD in parallel topology. The inset shows the measured presentative *I-V* curves. (**d**) Simulated output characteristics of the HEMT/RTD in series topology and the inset shows the measured typical *I-V* curves.

**Supplementary Fig. S23.** **Schematic diagram of an RTD-based oscillator circuit and oscillation spectrum.** (**a**) Schematic diagram of an RTD-based oscillator circuit and (**b**) high-frequency equivalent circuit. (**c**) S-parameters of the resonator cavity simulated by HFSS and fitted by ADS. (**d**) DC characteristics, (**e**) spectrum, (**f**) output voltage swing across the load resistance for NDR devices with different *ΔV.*
